# Adverse events of interest following influenza vaccination, a comparison of cell culture-based with egg-based alternatives: English sentinel network annual report paper 2019/20

**DOI:** 10.1016/j.lanepe.2021.100029

**Published:** 2021-01-13

**Authors:** Simon de Lusignan, Ruby S.M. Tsang, Gayatri Amirthalingam, Oluwafunmi Akinyemi, Julian Sherlock, Manasa Tripathy, Alexandra Deeks, Filipa Ferreira, Gary Howsam, F.D.Richard Hobbs, Mark Joy

**Affiliations:** a Nuffield Department of Primary Care Health Sciences, University of Oxford, Woodstock Road, Oxford OX2 6GG, United Kingdom; b Public Health England, 61 Colindale Avenue, London NW9 5EQ, United Kingdom; c Royal College of General Practitioners Research and Surveillance Centre, 30 Euston Square, London NW1 2FB, United Kingdom

**Keywords:** Influenza, Influenza vaccines, Adverse events of interest, Medical records systems, computerized, Sentinel surveillance

## Abstract

**Background:**

The cell-based quadrivalent influenza vaccine (QIVc) is now offered as an alternative to egg-based quadrivalent (QIVe) and adjuvanted trivalent (aTIV) influenza vaccines in the UK. While post-licensure studies show non-inferiority of cell-based vaccines, it is not known how its safety profile compares to other types of vaccines in real-world use.

**Methods:**

We conducted a retrospective cohort study using computerised medical records from the Royal College of General Practitioners (RCGP) Research and Surveillance Centre (RSC) sentinel network database. We used a self-controlled case series design and calculated the relative incidence (RI) of adverse events of interest (AEIs) over different risk periods. We then compared the RIs of AEIs within seven days of vaccination overall and between QIVc and QIVe in the 18–64 years age group, and between QIVc and aTIV in the ≥65 years age group.

**Findings:**

The majority of AEIs occurred within seven days of vaccination, and a seasonal effect was observed. Using QIVc as the reference group, QIVe showed similar incidence of AEIs whereas live attenuated influenza vaccine (LAIV) and aTIV had lower incidence of AEIs. In the stratified analyses, QIVe and aTIV were associated with a 16% lower incidence of AEIs in the seven days post-vaccination in both the 18–64 years and ≥65 years age groups.

**Interpretation:**

Routine sentinel network data allow comparisons of safety profiles of equally suitable seasonal influenza vaccines. The higher incidence of AEIs associated with QIVc suggest monitoring of several seasons would allow robust comparisons to be made.

**Funding:**

Public Health England.

**Research in context**

**Evidence before this study**

To summarise evidence on the safety profile of cell culture-based influenza vaccines, we conducted a search in MEDLINE on 16th October 2020 for journal articles using a combination of search terms including “cell culture-based”, “cell culture-derived”, “influenza vaccine”, “adverse event”, “adverse reaction”, and “safety”. We excluded articles on pandemic influenza vaccines and reviews, and limited the results to articles published in English between 2000 and 2020. We identified 18 relevant studies, of which 16 are reports of clinical trials and two are post-licensure safety surveillance studies. Trials that compared cell culture-based and egg-based trivalent influenza vaccines generally reported comparable safety and reactogenicity profiles across various age groups. Two trials that compared cell culture-based quadrivalent influenza vaccine (QIVc) with cell culture-based trivalent influenza vaccines found slightly more frequent solicited adverse events. Post-licensure safety surveillance studies did not find any concerning patterns of adverse events associated with cell culture-based influenza vaccines.

**Added value of this study**

This study provides the first comparison of incidence of adverse events of interest associated with QIVc and its egg-based alternatives using real-world evidence. The key finding here is that in age-stratified analyses, the recommended ‘equally suitable’ alternative to QIVc in the 18–64 years (QIVe) and ≥65 years (aTIV) age groups had a 16% lower incidence of AEIs in the seven days post-vaccination. Our post-hoc analysis also showed reactogenicity profiles differed between vaccine types and influenza seasons.

**Implications of all the available evidence**

Routine sentinel network data can provide real-world evidence for the safety of different types of vaccines. While earlier clinical trials showed comparable safety and reactogenicity profiles between cell culture-based and egg-based vaccines, our study using a much larger sample detected a higher incidence of adverse events of interest in those who received the cell culture-based quadrivalent influenza vaccine. Our findings highlight the importance of ongoing monitoring of adverse events of interest; the availability of longitudinal data would allow for more robust comparisons to be made.

Alt-text: Unlabelled box

## 1 Introduction

Cell culture-based influenza vaccines may have advantages over traditional egg-based vaccines. Firstly, it requires shorter production time, which allows for quicker scale-up of vaccine production in case of an influenza pandemic [1]. The cell culture-based vaccine also presents no risk to individuals who are allergic to eggs [2]. Most importantly, it circumvents concerns around the haemagglutinin mutations that occur during isolation, adaptation and propagation in eggs, which can alter viral antigenicity and are hypothesised to contribute to lower vaccine effectiveness (VE) [3,4].

Several VE studies have been conducted using real-world data from the United States, but the evidence is mixed. Two large retrospective cohort studies reported that the cell culture-based influenza vaccine (QIVc) was more effective in preventing hospital encounters related to influenza and certain respiratory events [5,6]. One study found QIVc provided better protection against only influenza B than egg-based influenza vaccines [7], while another found the egg-based quadrivalent influenza vaccine (QIVe) provided better protection against only A(H1N1)pdm09 than QIVc [8]. One retrospective cohort study and one test-negative case-control study reported no significant differences in effectiveness between QIVc and QIVe [9,10].

A cell culture-based quadrivalent influenza vaccine (Flucelvax® Tetra, Seqirus) was licensed for use in the United Kingdom (UK) for patients aged nine years and above in 2018, and was first recommended in the national influenza vaccination programme in the 2019/20 influenza season [11]. This QIVc is prepared from influenza virus propagated in Madin-Darby Canine Kidney (MDCK) cells. The Joint Committee on Vaccination and Immunisation (JCVI) considers it ‘equally suitable’ to the QIVe for those aged nine to 64 years in clinically at-risk and other eligible groups, and to the adjuvanted trivalent influenza vaccine (aTIV) in those aged ≥65 years [12].

More studies are needed to better evaluate the differences between QIVc and QIVe, yet there have not been any published studies to date that examine the safety of QIVc relative to other types of vaccines using real-world data. A better understanding of its safety profile will allow us to better weigh the risks and benefits associated with QIVc.

We conducted this study to: (1) calculate the relative incidence (RI) of adverse events of interest (AEIs) following seasonal influenza vaccination across all vaccine types, (2) compare RIs between QIVc and QIVe in at-risk adults aged 18 to 64 years, and (3) compare RIs between QIVc and aTIV in older adults aged ≥65 years.

## 2 Methods

### 2.1 Data source

We used the Royal College of General Practitioners (RCGP) Research and Surveillance Centre (RSC) sentinel network database. This is derived from pseudonymised extracts of computerised medical record (CMR) system data. The UK lends itself to this type of study as it has registration-based primary care (one patient registers with a single general practice) and CMRs have been in routine use in the UK for over 20 years. The RCGP RSC was established in 1957, and has been involved in influenza monitoring and assessing influenza vaccine effectiveness since 1967 [13]. The scheme includes practices recruited to be representative of the population [14]. At the time of this study, the RCGP RSC included around 5 million patient records from >500 practices across England; but has more recently expanded considerably in response to the COVID-19 pandemic [15].

We included all patients who had received a seasonal influenza vaccination between 1st September 2019 and 30th April 2020, but excluded those who were aged over 100 years at the time of vaccination and those who received monovalent pandemic influenza vaccines. Patients who attended practices involved in other enhanced surveillance programmes were also excluded. From our experience, this more active surveillance increased awareness and recording at the practices, and captured more adverse events such as local minor reactions for which vaccines may not have sought medical attention otherwise [16], which may distort comparisons between types or brands of vaccine and between years. Patients were followed up retrospectively for a list of adverse events of interest (AEIs) following vaccination as pre-specified by the European Medicines Agency (EMA) [17]. We had used a set of Read codes that matched the EMA's list to identify AEIs in earlier studies as well as three years of observation for a vaccine manufacturer, and this set of codes has been updated to SNOMED clinical terms (the UK's new clinical terminology) for this study. The AEIs identified include common conditions which may not be causally related to the vaccine, any signal is inferred from a change in incidence in the seven days following vaccination.

We extracted the following data: age, sex, self-reported ethnicity using an ontology to maximise data capture [18], socioeconomic status using Index of Multiple Deprivation (IMD) quintiles, vaccination date, vaccine type, vaccine manufacturer, AEI date, AEI type, and dates of registration and deregistration at the practice. IMD is derived from post code of the patient at the individual level at the point of data extraction, the post code is not retained [19].

Finally, we compared the rates of AEIs with the previous season, where available. We did this because in previous research we saw year-on-year changes in AEIs [20].

### 2.2 Statistical analyses

We used the self-controlled case series (SCCS) design [21,22] for this study. The SCCS method is a case-only method that compares the rate of events during pre-defined exposure risk periods with the rate of events during all remaining time in the observation period (i.e. baseline risk periods) within individuals. The major advantage of this method is that it eliminates potential time-invariant confounding effects of between-person characteristics, as each individual acts as their own control.

We conducted three separate SCCS models to investigate the incidence of AEIs following seasonal influenza vaccination, with the observation period defined as 1st September 2019 to 30th April 2020 in all models. In Model 1, the exposure risk periods were days −7 to −1, days 0 to 6, days 7 to 13, and days 14 to 45 where day 0 is the day of vaccination. Given the seasonal variation in the incidence of some of the events of interest, we incorporated an additional variable with the observation period divided into 8 seasonal periods: days 0 to 29, days 30 to 59, days 60 to 89, days 90 to 119, days 120 to 149, days 150 to 179, days 180 to 209, days 210 to 241 where day 0 is the beginning of the influenza season. We calculated the RI of AEIs following vaccination for the different exposure risk periods and different seasonal periods. In Model 2, we modelled modification effects of vaccine type by including an interaction term. Models 3 and 4 were stratified analyses in which we compared the RIs of AEIs between QIVc and QIVe in those aged 18 to 64 years and between QIVc and aTIV in those aged ≥65 years respectively by adding an interaction term in the models. In Models 2–4 we focused only on the exposure risk period of days 0 to 6, as previous studies showed the risk of AEIs were highest in this period [20].

All statistical analyses were conducted in R version 3.4.4 [23], with the following packages: tidyverse version 1.2.1 [24], SCCS version 1.1 [25], lubridate version 1.7.4 [26], tableone version 0.10.0 [27]. Graphical output was generated using packages ggplot2 version 3.1.1 [28] and ggthemes version 4.1.0 [29].

### 2.3 Ethical considerations

All potentially identifiable data were pseudonymised as close to source as possible and not made available to researchers; data were not extracted for patients who opted out of data sharing. All data were stored and processed at the RCGP RSC secure data and analytics hub, the University of Surrey. According to the Health Research Authority and Medical Research Council Regulatory Support Centre's online decision tool, this study falls under the category of service evaluation and does not require further ethical review. This study was approved by the RCGP.

### 2.4 Role of the funding source

The RCGP RSC's principal funder is Public Health England (PHE). PHE had no role in study design; data collection, analysis, or interpretation; writing of the report; or the decision to submit it for publication. No specific funding was allocated for the writing of this annual report paper, which focusses on a topic issue, in this case whether the introduction of cell culture-based manufactured influenza vaccine would be associated with any difference in the pattern of adverse events of interest. The corresponding author had full access to all the data in the study and final responsibility for the decision to submit for publication.

## 3 Results

### 3.1 Study participants

A total of 1,108,632 patients in the RCGP RSC network who met the inclusion criteria received a seasonal influenza vaccine in the 2019/20 season. This cohort had a mean age of 56 years and a slight female preponderance (54.5% female); a large majority (72.9%) was of white ethnicity. Detailed demographic characteristics of the cohort are presented in Table 1.

**Table 1:** Demographic characteristics of all recipients of seasonal influenza vaccines in the 2019/20 season in the RCGP RSC network (*n* = 1,108,632).

	Mean±SD / n (%)
Age (years)	56.04±26.79
Sex	
Female	604,048 (54.5%)
Male	504,584 (45.5%)
Ethnicity	
White	808,086 (72.9%)
Asian	53,538 (4.8%)
Black	21,395 (1.9%)
Mixed	9,750 (0.9%)
Other	6,665 (0.6%)
Missing	209,198 (18.9%)
Index of Multiple Deprivation (IMD) Quintile	
1 – most deprived	148,646 (13.4%)
2	182,643 (16.5%)
3	217,221 (19.6%)
4	247,451 (22.3%)
5 – least deprived	285,674 (25.8%)
Missing	26,997 (2.4%)

**Fig. 1:**
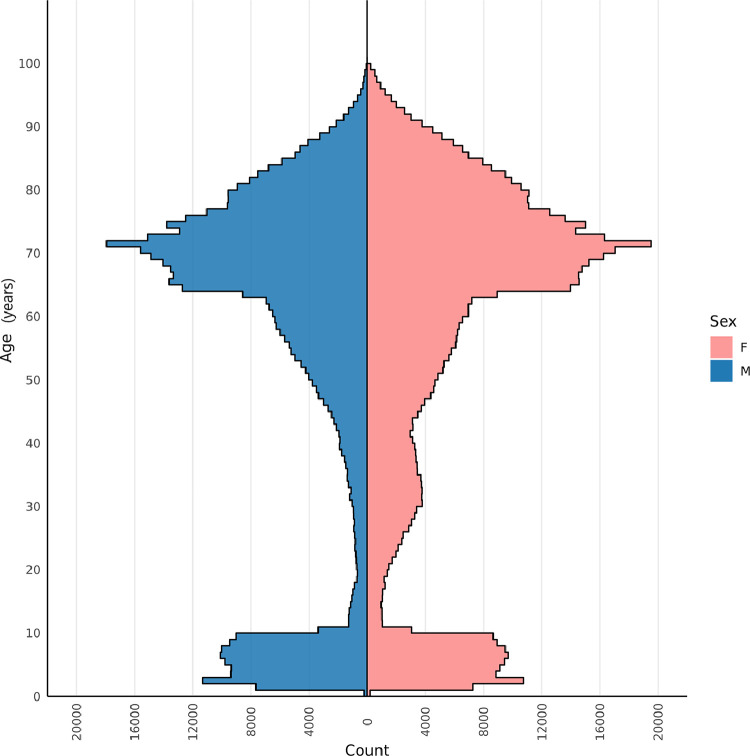
Age-sex profile for all seasonal influenza vaccine recipients in the RCGP RSC network in the 2019/20 season.

The age-sex profile (Fig. 1) shows a peak in the older age group, with the sharp increase beginning from 64 years, and reaching the peak at 71 years. Children between the ages of one and ten years are the next biggest group of vaccines. In addition, there is a small increase in numbers of women aged between 20 and 40 years, probably reflecting the recommendation for vaccination in pregnant women.

### 3.2 Types of vaccines

We were able to identify 98.6% of the vaccines administered. There were six categories of seasonal influenza vaccines in our dataset, comprising the adjuvanted trivalent influenza vaccine (aTIV), quadrivalent live attenuated influenza vaccine (LAIV), QIV (unspecified whether cell- or egg-based), QIVc, QIVe, trivalent influenza vaccine (TIV) and high-dose trivalent influenza vaccine (TIV-HD) (Table 2). We excluded the latter two categories from the analysis due to low numbers. We further excluded the QIV (unspecified) category and conducted complete case analysis in the stratified models.

**Table 2:** Total number of different types of vaccines by age group.

	0–1yr	2–17yr	18–64yr	≥65yr
aTIV	0	267	10,073	616,648
LAIV	56	123,094	175	5
QIV	1	108	10,345	2,769
QIVc	5	305	26,687	29,384
QIVe	765	7,052	260,574	4,051
TIV	0	20	228	356
TIV-HD	0	0	2	4

Note. yr: years of age; aTIV: adjuvanted trivalent influenza vaccine; LAIV: live attenuated influenza vaccine; QIV: quadrivalent influenza vaccine; QIVc: cell culture-based quadrivalent influenza vaccine; QIVe: egg-based quadrivalent influenza vaccine; TIV: trivalent influenza vaccine; TIV-HD: high-dose trivalent influenza vaccine.

### 3.3 Incidence of AEIs

We observed AEIs in every EMA category, with the most commonly reported being arthropathy, cough and rash (Table 3). Of all AEIs, the ones that were most frequently reported within seven days of vaccination were wheezing, nasal congestion and cough. More severe conditions such as Guillain-Barré syndrome (*n* = 1) and anaphylaxis (*n* = 6) were rare within seven days of vaccination.

**Table 3:** Total number of reported adverse events of interest across the 2019/20 season and within 7 days of vaccination.

	Within influenza season		Within 7 days of vaccination			
	Total events	Unique individuals	Total events	Unique individuals	7-day cumulative incidence (events per 100,000 doses)	Proportion of AEIs within 7 days of vaccination
Fever / pyrexia						
Fever (unspecified)	10,436	8,763	325	315	29.32	0.0311
Mild fever (<=38.5 °C)	16,491	15,011	470	465	42.39	0.0285
Moderate fever (38.6–39.5 °C)	2,258	2,199	81	81	7.31	0.0359
High fever (>39.5 °C)	312	309	11	11	0.99	0.0353
Gastrointestinal						
Decreased appetite	3,367	2,859	135	133	12.18	0.0401
Diarrhoea	21,196	14,250	677	629	61.07	0.0319
Nausea	5,801	4,070	193	181	17.41	0.0333
Vomiting	8,591	6,405	248	238	22.37	0.0289
General non-specific symptoms						
Drowsiness	849	684	22	22	1.98	0.0259
Fatigue	16,664	11,998	942	909	84.97	0.0565
Headache	22,654	14,906	976	937	88.04	0.0431
Irritability	261	216	8	8	0.72	0.0307
Malaise	8,700	6,479	322	305	29.04	0.0370
Local symptoms (i.e. local erythema)	362	148	8	8	0.72	0.0221
Musculoskeletal						
Arthropathy	162,996	88,236	8,614	8,316	776.99	0.0528
Muscle aches / myalgia	13,548	5,095	560	538	50.51	0.0413
Neurological						
Bell's palsy	601	375	20	18	1.80	0.0333
Guillain-Barré syndrome (GBS)	61	35	1	1	0.09	0.0164
Peripheral tremor	2,822	1,950	153	148	13.80	0.0542
Seizure / febrile convulsions	7,396	3,862	348	337	31.39	0.0471
Rash	34,771	24,854	1,558	1,517	140.53	0.0448
Respiratory / miscellaneous						
Conjunctivitis	8,482	7,200	339	336	30.58	0.0400
Coryza	1,348	1,260	73	73	6.58	0.0542
Cough	90,246	63,019	5,185	5,080	467.69	0.0575
Epistaxis	5,932	4,231	209	203	18.85	0.0352
Hoarseness	2,329	1,704	120	119	10.82	0.0515
Nasal congestion	2,067	1,741	120	120	10.82	0.0581
Oropharyngeal pain	15,281	12,137	474	460	42.76	0.0310
Rhinorrhoea	1,131	973	55	55	4.96	0.0486
Wheezing	10,542	8,629	1,152	114	103.91	0.1093
Sensitivity / anaphylaxis						
Anaphylactic reactions	281	196	7	6	0.63	0.0249
Facial oedema	82	77	3	3	0.27	0.0366
Hypersensitivity reactions	7,662	6,585	416	411	37.52	0.0543

Note. The “total events” columns are the frequencies of events of interest within the indicated periods, including repeated consultations with the same individual. The “unique individuals” columns are counts of individuals who presented with these events of interest within the indicated periods, regardless of whether there were repeated consultations.

The four pre-specified risk periods were associated with different RIs of AEIs, with the majority of AEIs occurring in the seven days post-vaccination (Table 4). The seven days post-vaccination showed a doubling of risk of AEIs (RI=2.05, 95% CI 2.02–2.08) whereas the risk periods of days 7 to 13 and days 14 to 45 were associated with only a marginally increased RI. The seven days leading up to vaccination was associated with a 6% lower RI of AEIs (RI=0.94, 95% CI 0.92–0.96), reflecting the “healthy vaccine” effect that has previously been reported [20].

**Table 4:** Model 1: Relative incidence of adverse events of interest in various risk periods and seasonal periods.

	RI	95% CI	*p*
*Exposure risk period (with date of vaccination as day 0)*			
Days −7 to −1	0.94	0.92–0.96	<0.001
Days 0 to 6	2.05	2.02–2.08	<0.001
Days 7 to 13	1.04	1.02–1.06	<0.001
Days 14 to 45	1.03	1.01–1.04	<0.001
*Time from start of the influenza season (reference: Days 0 to 29)*			
Days 30 to 59	1.00	0.99–1.02	0.694
Days 60 to 89	1.05	1.03–1.06	<0.001
Days 90 to 119	0.90	0.89–0.92	<0.001
Days 120 to 149	1.12	1.11–1.13	<0.001
Days 150 to 179	1.11	1.09–1.12	<0.001
Days 180 to 209	0.88	0.86–0.89	<0.001
Days 210 to 241	0.57	0.56–0.58	<0.001

In addition, we found a seasonal pattern in AEIs (Table 4). Using the first 30 days of the season as reference, the third, fifth and sixth seasonal periods showed an increase in AEIs (RI=1.05, 95% CI 1.03–1.06; RI=1.12, 95% CI 1.11–1.13; and RI=1.11, 95% CI 1.09–1.12 respectively), whereas the fourth, seventh and eighth seasonal periods showed a decrease in AEIs (RI=0.90, 95% CI 0.89–0.92; RI=0.88, 95% CI 0.86–0.89; and RI=0.57, 95% CI 0.56–0.58 respectively). The periods that showed decreases in consultations for AEIs roughly correspond to when the UK went into national lockdown due to the COVID-19 pandemic (announced on 23rd March 2020 and continued beyond 30th April 2020).

### Incidence of AEIs by vaccine type

We added an interaction term to compare the incidence of AEIs associated with the different vaccine types across the entire cohort. The results showed that QIVc and QIVe were associated with a similar incidence of AEIs, whereas LAIV and aTIV were associated with 43% and 26% lower RIs than QIVc respectively (Table 5).

**Table 5:** Model 2: Relative incidence of adverse events of interest and interaction term for vaccine type.

	RI	95% CI	*p*
*Exposure risk period (with date of vaccination as day 0)*			
Days 0 to 6	2.42	2.30–2.55	<0.001
*Time from start of influenza season (reference: Days 0 to 29)*			
Days 30 to 59	1.00	0.99–1.02	0.557
Days 60 to 89	1.05	1.04–1.07	<0.001
Days 90 to 119	0.91	0.90–0.92	<0.001
Days 120 to 149	1.12	1.11–1.14	<0.001
Days 150 to 179	1.11	1.09–1.12	<0.001
Days 180 to 209	0.88	0.86–0.89	<0.001
Days 210 to 241	0.57	0.56–0.58	<0.001
*Vaccination type (reference: QIVc)*			
QIVe	0.96	0.90–1.02	0.148
LAIV	0.57	0.53–0.61	<0.001
aTIV	0.74	0.70–0.78	<0.001

Note. QIVc: cell culture-based quadrivalent influenza vaccine; egg-based quadrivalent influenza vaccine; LAIV: live attenuated influenza vaccine; aTIV: adjuvanted trivalent influenza vaccine.

### 3.4 Stratified analyses

We compared the incidence of AEIs associated with QIVc and QIVe in adults aged 18 to 64 years by incorporating an interaction term to the model. The results showed that relative to QIVc, QIVe was associated with 16% lower RI of AEIs (RI=0.84, 95% CI 0.78–0.91) (Table 5).

Similarly, we conducted a stratified analysis in older adults aged ≥65 years to compare the incidence of AEIs associated with QIVc and aTIV. The results showed that relative to QIVc, aTIV was associated with 16% lower RI of AEIs (RI=0.84, 95% CI 0.78–0.91) (Table 6).

**Table 6:** Model 3: Relative incidence of adverse events of interest and interaction term for vaccine type in adults aged 18 to 64 years.

	RI	95% CI	*p*
*Exposure risk period (with date of vaccination as day 0)*			
Days 0 to 6	2.84	2.63–3.06	<0.001
*Time from start of influenza season (reference: Days 0 to 29)*			
Days 30 to 59	0.97	0.94–0.99	0.006
Days 60 to 89	0.98	0.95–1.00	0.069
Days 90 to 119	0.83	0.81–0.85	<0.001
Days 120 to 149	1.07	1.04–1.10	<0.001
Days 150 to 179	1.05	1.02–1.07	<0.001
Days 180 to 209	0.88	0.86–0.90	<0.001
Days 210 to 241	0.59	0.57–0.60	<0.001
*Vaccination type (reference: QIVc)*			
QIVe	0.84	0.78–0.91	<0.001

Note. QIVc: cell culture-based quadrivalent influenza vaccine; QIVe: egg-based quadrivalent influenza vaccine.

**Table 7:** Model 4: Relative incidence of adverse events of interest and interaction term for vaccine type in older adults aged ≥65 years.

	RI	95% CI	*p*
*Exposure risk period (with date of vaccination as day 0)*			
Days 0 to 6	2.13	1.97–2.30	<0.001
*Time from start of influenza season (reference: Days 0 to 29)*			
Days 30 to 59	1.00	0.98–1.01	0.556
Days 60 to 89	1.04	1.02–1.06	<0.001
Days 90 to 119	0.89	0.88–0.91	<0.001
Days 120 to 149	1.16	1.14–1.18	<0.001
Days 150 to 179	1.11	1.09–1.13	<0.001
Days 180 to 209	0.86	0.84–0.87	<0.001
Days 210 to 241	0.59	0.58–0.60	<0.001
*Vaccination type (reference: QIVc)*			
aTIV	0.84	0.78–0.91	<0.001

## 4 Discussion

### 4.1 Key findings

In this study, we examined the incidence of AEIs following seasonal influenza vaccination, and compared that of QIVc versus QIVe in clinically at-risk adults aged 18 to 64 years, and that of QIVc and aTIV in older adults aged ≥65 years. In line with data from earlier influenza seasons [20], we observed AEIs mainly occur within seven days of vaccination. The seasonal pattern observed was different to that of previous seasons; the RIs of AEIs was significantly lower in the last two seasonal periods, which was expected with the national lockdown in place at the time. This sharp decline in the number of consultations for respiratory conditions during that period can also be seen in our COVID-19 Observatory weekly return (orchid.phc.ox.ac.uk/index.php/cov-19/), and a recent analysis of primary care consultations in older adults similarly reported a reduction in total consultations [30].

Overall, the incidence of AEIs in the seven days post-vaccination did not appear to differ among patients who received QIVc or QIVe, but aTIV and LAIV were associated with lower RIs of AEIs. This general pattern was observed in the 2018/19 season too, with QIVe associated with higher incidence of AEIs relative to aTIV and LAIV [31]. This may partly be due to differences in reactogenicity in the different age groups, so we also conducted stratified analyses between two equally suitable vaccines within an age group.

In the stratified analyses, we found that QIVc was associated with higher RIs of AEIs within seven days of vaccination when compared to QIVe in adults aged 18 to 64 years and to aTIV in older adults aged ≥65 years. This is an unanticipated finding, as phase III trials showed the safety profile of QIVc was similar to two comparator TIVc, with only slightly higher incidence of solicited local AEIs [32,33], and post-licensure studies have repeatedly shown that the safety of QIVc is similar to cell-based TIV (TIVc), which in turn is similar to egg-based TIV [34].

We also explored whether the different types of vaccines were associated with different categories of AEIs. We found that the incidence of AEIs in most EMA categories were comparable between the four vaccines. The only category in which the incidence of AEIs associated with QIVc was evidently higher than the other vaccines was musculoskeletal symptoms (Figure S1 in the Supplementary); specifically, arthropathy accounted for a large part of this higher incidence.

It is possible that the differences in incidence of AEIs partly reflect health disparities between demographic groups. In the stratified analyses, there appears to be some differences in the age, sex, ethnicity and IMD quintile distributions between the vaccine groups (see Table S1 in Supplementary). Within the 18 to 64 years age group, a slightly greater proportion of QIVc recipients were female, from an ethnic minority and in the least deprived quintile compared to the QIVe group. For the ≥65 years age group, QIVc recipients were older, a greater proportion of them were female, from an ethnic minority as well as in the least deprived quintile compared to the aTIV group. Earlier studies that examined sex differences in the safety of seasonal influenza vaccines have reported higher rates of AEIs in females [35]. Greater pain sensitivity in females, hypersensitivity reactions, route of administration and hormonal factors were suggested to contribute to such differences [36].

### 4.2 Strengths and limitations

To our knowledge, this is the first study to evaluate the relative safety of QIVc versus other types of vaccines using real-world data. The use of data from a large nationally representative sentinel network like the RCGP RSC provides us with adequate sample sizes to compare the safety of ‘equally suitable’ vaccines in stratified analyses. These models yielded important insights that have not previously been reported in clinical trials or other post-licensure studies. Our cohort is large and nationally representative in terms of demographics and clinical outcomes, so we believe the findings could be generalisable to other countries with a similar demographic composition.

Given 2019/20 is the first influenza season in which the QIVc was used, only a small proportion of patients received the QIVc (8.06% in the 18 to 64 years group, and 4.51% in the ≥65 years group). While our data are as good as they get in primary care, we acknowledge that incomplete data is an important limitation, for instance where vaccinations took place outside the general practice and the practice had not been notified [37], or where information on vaccine brand or batch was not recorded. We make enormous efforts to capture complete data, including asking individual sentinel network practices to inform us in advance of the brand and batch of vaccines they have ordered at the start of each influenza season. Moreover, as the study is observational in nature, there is some degree of demographic difference between the vaccine groups. In particular, sex, ethnic and socioeconomic disparities in health are well documented, and we are unable to rule out potential confounding effects of these factors. Last but not least, reduced vaccinations as well as consultations due to the COVID-19 pandemic may have had an effect on our data, which could reduce the generalizability of these findings to other influenza seasons.

### 4.3 Future research

This study provides preliminary evidence that QIVc may be associated with a higher incidence of AEIs. Further evaluation of its safety using data from different countries and future influenza seasons would be beneficial. It would also be informative to explore the specific events of interest associated with the different types of vaccines as well as their severity. With the likely co-circulation of influenza and COVID-19 in the 2020/21 season, the safety of seasonal influenza vaccines in patients who have recovered from COVID-19, particularly those who are experiencing post-acute COVID-19 (‘long COVID’) [38], remain to be investigated. In addition, when a COVID-19 vaccine becomes available, the safety of concomitant administration of seasonal influenza vaccines with the COVID-19 vaccine would also need to be assessed.

## 5 Conclusion

Safety profiles of recommended seasonal influenza vaccines, particularly those of equally suitable vaccines within an age group, can be compared using routine sentinel network data. We report that QIVc is associated with a higher incidence of AEIs compared to QIVe and aTIV in the 18 to 64 years and ≥65 years age groups respectively. Despite the advantages that cell culture-based influenza vaccines may have, the higher risk of AEIs observed in patients who received QIVc suggest further monitoring for several influenza seasons would allow for robust comparisons to be made that will inform future seasonal influenza vaccination recommendations.

## Contributors

SdeL conceived the study. SdeL, OA, MJ contributed to study design. JS performed data extraction. RSMT performed data analysis. RSMT and SdeL contributed to manuscript preparation. All authors contributed to elements of the study, manuscript revisions, and approved the final version of the manuscript.

## Data sharing statement

The RCGP RSC dataset can be accessed by researchers, approval is on a project-by-project basis (www.rcgp.org.uk/rsc). Ethical approval by an NHS Research Ethics Committee is needed before any data release/other appropriate approval. Researchers wishing to directly analyse patient-level pseudonymised data will be required to complete information governance training and work on the data from the secure servers at the University of Oxford. Patient-level data cannot be taken out of the secure network.

## Declaration of Competing Interest

SdeL is the Director of the RCGP RSC, its principal funder is Public Health England, who had no direct involvement with this publication. SdeL has received funding through his University for studies from AstraZeneca, Daiichi Sankyo, Eli Lilly, Sanofi, GSK, MSD, Seqirus and Takeda; and has been a member of advisory boards for Seqirus and Sanofi. The GSK funding included monitoring adverse events of interest for their quadrivalent influenza vaccine for three seasons 2015-2018. FDRH has received personal fees from Boehringer Ingelheim, Novartis and Pfizer. All other authors have no competing interests to declare.

## Appendix Supplementary materials

Image, application 1
