# Supplementary material for: Adverse events of interest following influenza vaccination, a comparison of cell culture-based with egg-based alternatives: English sentinel network annual report paper 2019/20
Source: Lancet Reg Health Eur. 2021 Jan 13;2:100029. doi: 10.1016/j.lanepe.2021.100029 (PMC8454842; doi:10.1016/j.lanepe.2021.100029)

Supplementary Material

Table S1. Comparison of demographics characteristics between vaccine groups in Models 2 and 3 [mean±SD / n (%)].

**Model 2**	**QIVc (n=11,462)**	**QIVe (n=130,756)**
Age (years)	48·89±12·11	48·85±12·12
Sex		
Female	7,707 (67·24%)	85,656 (65·51%)
Male	3,755 (32·76%)	45,100 (34·49%)
Ethnicity		
White	8,081 (70·50%)	97,454 (74·53%)
Asian	845 (7·37%)	10,660 (8·15%)
Black	500 (4·36%)	3,576 (2·73%)
Mixed	180 (1·57%)	1,209 (0·92%)
Other	269 (2·35%)	1,180 (0·90%)
Missing	1,587 (13·85%)	16,677 (12·75%)
IMD Quintile		
1 – most deprived	1,987 (17·34%)	28,399 (21·72%)
2	2,287 (19·95%)	25,032 (19·14%)
3	1,993 (17·39%)	25,436 (19·45%)
4	2,210 (19·28%)	25,823 (19·75%)
5 – least deprived	2,879 (25·12%)	23,302 (17·82%)
Missing	106 (0·92%)	2,764 (2·11%)
**Model 3**	**QIVc (n=13,795)**	**aTIV (n=291,766)**
Age (years)	77·06±7·94	76·80±7·06
Sex		
Female	8,469 (61·39%)	174,191 (59·70%)
Male	5,326 (38·61%)	117,575 (40·30%)
Ethnicity		
White	9,650 (69·95%)	235,649 (80·77%)
Asian	589 (4·27%)	8,751 (3·00%)
Black	360 (2·61%)	2,458 (0·84%)
Mixed	87 (0·63%)	743 (0·25%)
Other	158 (1·15%)	780 (0·27%)
Missing	2,951 (21·39%)	43,385 (14·87%)
IMD Quintile		
1 – most deprived	1,807 (13·10%)	33,134 (11·36%)
2	2,104 (15·25%)	43,708 (14·98%)
3	2,158 (15·64%)	62,395 (21·39%)
4	2,472 (17·92%)	72,087 (24·71%)
5 – least deprived	5,224 (37·87%)	75,629 (25·92%)
Missing	30 (0·22%)	4,813 (1·65%)

Note. IMD: Index of Multiple Deprivation; QIVc: cell culture-based quadrivalent influenza vaccine; QIVe: egg-based quadrivalent influenza vaccine; aTIV: adjuvanted trivalent influenza vaccine

Figure S1. Incidence of adverse events of interest within 7 days of vaccination by European Medicines Agency categories and vaccine type in the 2018/19 and 2019/20 seasons.

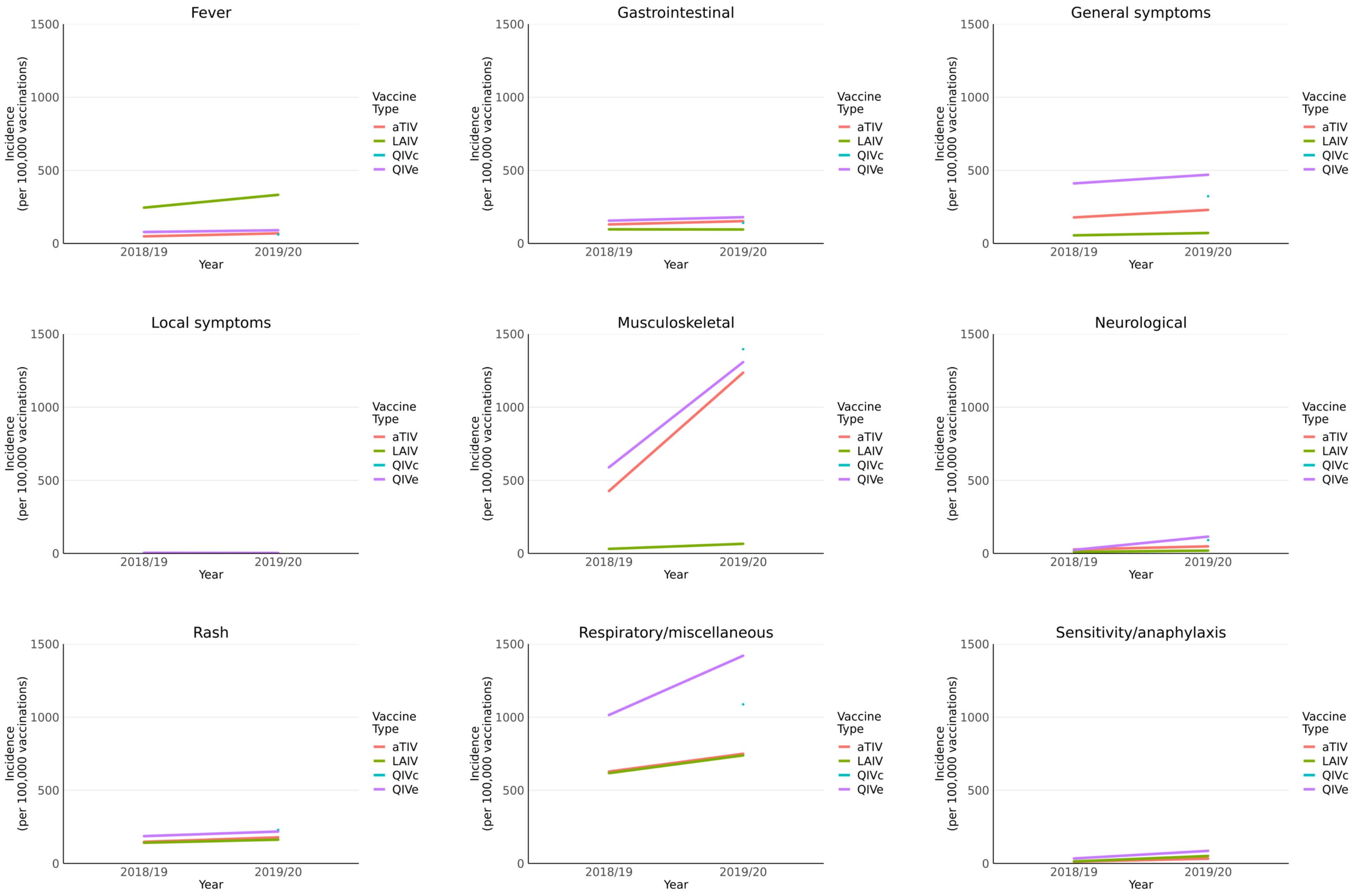

Supplement: Supplementary file 1 [file mmc1.pdf]
